# Use of multiple metrics to assess antibiotic use in Italian children’s hospitals

**DOI:** 10.1038/s41598-021-83026-1

**Published:** 2021-02-11

**Authors:** Carmen D’Amore, Marta Luisa Ciofi degli Atti, Carla Zotti, Rosa Prato, Giuliano Guareschi, Raffaele Spiazzi, Gaetano Petitti, Maria Luisa Moro, Massimiliano Raponi

**Affiliations:** 1 Clinical Pathways and Epidemiology Unit, Bambino Gesù Children’s Hospital, Rome, Italy; 2 Department of Public Health and Pediatrics, University of Turin, Turin, Italy; 3 Department of Medical and Surgical Sciences, University of Foggia, Ospedale “D’Avanzo” Policlinico Riuniti, Foggia, Italy; 4 Ospedale Infantile Regina Margherita - AOU Città Della Salute E Della Scienza Di Torino, Torino, Italy; 5 Ospedale dei Bambini - ASST Degli Spedali Civili di Brescia, Brescia, Italy; 6 Azienda Ospedaliero Universitaria Consorziale Policlinico di Bari Ospedale Pediatrico “Giovanni XXIII”, Bari, Italy; 7 Regional Health and Social Agency, Emilia-Romagna Region, Italy; 8 Medical Direction, Bambino Gesù Children’s Hospital, Rome, Italy

**Keywords:** Paediatrics, Drug therapy, Antimicrobial therapy

## Abstract

Quantification of antibiotic utilization is an essential component of antibiotic stewardship programs. In this multicentric study, we used different metrics to evaluate inpatient antibiotic use in children. The study objectives were to describe point prevalence of antibiotic use by indication and patient characteristics, to evaluate DOTs, LOTs and PDDs, and to compare PDDs to DDDs, which assume average maintenance dose per day in adults. All children hospitalized on the days of the study were included. Trained personnel collected demographic and clinical data from patients’ clinical records. We recorded information about antibiotics administered on the date of data collection, and in the previous 30 days of hospitalization. Of 810 patients, 380 (46.9%; CI 95%: 43.4–50.4) received one or more antibiotics; prevalence of use was 27.0% for prophylaxis (219/810), and 20.7% (168/810) for treatment. Overall, 587 drugs were issued to the 380 patients receiving antibiotics (1.5 antibiotic per patient). When considering treatments, DOT and LOT per 100 patient-days were 30.5 and 19.1, respectively, resulting in a DOT/LOT ratio of 1.6. PDDs increased with age and approached DDDs only in children aged ≥ 10 years; the ratio between PDDs estimated in children aged ≥ 10 years and in 0–11 month-old infants ranged from 2 for sulfamethoxazole and trimethoprim, to 25 for meropenem. Our results confirm that DOT, LOT and PDD are better alternatives to DDD in children.

## Introduction

Improving the use of antibiotics is an important patient safety and public health issue, as well as a global priority^1^.The misuse of antibiotics, in fact, has contributed to the growing problem of antibiotic resistance, which has become one of the most serious and growing public health threats^1,2^. Children represent an excellent population for the selection of resistant bacterial pathogens after recent antimicrobial use^3,4^. Therefore, the investigation of antibiotic consumption in the pediatric population is crucial, especially in the hospital setting where the risk of the emergence and transmission of bacterial resistance is dramatically increased^3^.

A growing body of evidence demonstrates that hospital based antibiotic stewardship programs (ASPs) can both optimize the treatment of infections and reduce adverse events associated with antibiotic use^5–7^. Quantification of antibiotic utilization is an integral part of ASPs. Hospital antibiotic consumption is commonly measured by calculating the daily defined doses (DDDs), as defined by the World Health Organization Collaborating Center for Drug Statistics Methodology, divided by a denominator indicating clinical activity (e.g. DDDs per 100 patient-days)^8,9^. DDD is calculated as the assumed average maintenance dose per day for a drug used for its main indication in adults. Even if DDDs allow the comparison of drug use between different settings and between different drugs, they do not reflect the dosage actually used for individual patients or specific patient populations^10^, and their use is not recommended in children^11^. To overcome these issues, in-hospital antibiotic use in children is frequently estimated by conducting point-prevalence surveys (PPS). Although the PPS design has been extensively used to evaluate antibiotic use, it cannot capture treatment duration. Other antibiotic consumption indicators are days of therapy (DOTs), length of therapy (LOTs) and prescribed daily doses (PDDs). DOTs are computed summing up the duration (in days) of each antibacterial drug received for treatments. LOTs are the number of days that a patient receives an antibacterial drug irrespective of the number of different drugs. DOTs and LOTs give an accurate estimation of polydrug therapy and duration of therapy, respectively; since they do not consider whether a nonstandard dose was given^12^, they could be considered as accurate metrics of drug use for children. The PDD provides the average daily amount of a drug that is actually prescribed^13^. In this multicentric study, we adopted different metrics to evaluate inpatient antibiotic use in hospitalized children. The study objectives were to describe point prevalence of antibiotic use by indication and patient characteristics, to estimate DOTs, LOTs and PDDs as additional metrics to quantify antibiotic use, and to compare PDDs to DDDs by antibiotic and age group.

## Results

### Point prevalence of antibiotic use

In our study, we included 810 children. Patients were mostly males (n = 422, 52%), aged ≥  10 years years (n = 233, 29%) and with a length of hospital stay ≤ 7 days (n = 458, 57%) (Table 1). About 38% of the patients (306/810) had at least one chronic disease; the most frequent chronic diseases were congenital heart diseases (70/306; 23%), genetic syndromes (38/306; 12%), chronic respiratory diseases (22/306; 7%), neuromuscular (19/306; 6%), and chronic renal diseases (18/306; 6%). Two hundred and thirty-one patients (29%) underwent at least one surgical procedure during hospitalization, for a total of 331 surgical procedures. The majority of patients (450/810; 56%), were admitted to medical units, 32% (261/810) to surgical units and 12% (99/810) to intensive care units (Table 1).

**Table 1:** Characteristics of patients receiving therapeutic and prophylactic antimicrobials in Italian Pediatric Hospitals, 2016.

	N. patients with at least 1 prescription	N. total patients	Prevalence % (CI 95%)	*P* value
**Sex**				
Female	162	379	50.9 (46.1–55.8)	0.02
Male	215	422	42.7 (37.7–47.9)	
Missing	3	9	–	
**Age**				
0–11 months	47	132	35.6 (27.5–44.4)	< 0.001
12–23 months	40	123	32.5 (24.4–41.6)	
2–4 years	100	182	54.9 (47.4–62.3)	
5–9 years	74	139	53.2 (44.6–61.7)	
≥ 10 years	119	233	51.1 (44.5–57.7)	
Missing	1	1	–	
**Length of hospital stay (days)**				
≤ 7	211	458	46.1 (41.4–50.8)	0.5
08–30	108	215	50.2 (43.4–57.1)	
> 30	61	137	44.5 (36.0–53.3)	
**Ward**				
Medical	196	450	43.6 (38.9–48.3)	0.1
Surgical	133	261	50.9 (44.7–57.2)	
Intensive care	51	99	51.5 (41.3–61.7)	
**Surgical procedure during present hospitalization**				
Yes	148	231	64.1 (57.5–70.3)	< 0.001
No	226	563	40.1 (36.1–44.3)	
Missing	6	16	–	
**Comorbidities**				
Yes	128	306	41.8 (36.2–47.6)	
No	242	482	50.2 (45.7–54.8)	0.02
Missing	10	22	–	
**Participating centre**				
1	237	460	51.5 (46.8–56.2)	< 0.001
2	52	154	33.8 (26.4–41.8)	
3	23	35	65.7 (47.8–80.9)	
4	68	161	42.2 (34.5–50.3)	
Total	380	810	46.9 (43.4–50.4)	

Of the 810 patients, 380 (46.9%; CI 95%: 43.4–50.4) received one or more antibiotics. The proportion of patients receiving antibiotics significantly varied by hospital, age, sex, comorbidities and surgical procedures. In particular, the prevalence of use was significantly higher in patients aged ≥ 2 years compared to those in the first year of life, in males with respect to females, in patients without comorbidities compared to those who had at least one chronic disease, and in patients who underwent at least one surgical procedure (Table 1).

Prophylaxis was the main indication for antibiotic use (217/380; 57.1%). Children receiving antibiotics for surgical or medical prophylaxis accounted for 14.3% (116/810) and 12.7% (103/810) of the hospitalized patients, respectively. Surgical prophylaxis lasted more than 24 h in 76.7% of the patients (89/116). Patients treated for CAIs were 13.2% (107/810) of children, followed by patients treated for HAIs (61/810; 7.5%).

Overall, 587 drugs were issued to the 380 patients receiving antibiotics (1.5 antibiotic per patient), mainly administered intravenously (465/587; 79.2%). Prescriptions for treatments were 42.8% (251/587); of those, the majority of antibiotics was administered for CAIs (147/251; 58.6%), and the remaining for HAIs (104/251; 41.4%). Overall, microbiological cultures were performed before starting the antibiotic therapy in 54.5% of the treatments (137/251). In 23.1% of the prescriptions (58/251), the results of the antibiogram were available and the antibiotic therapy could be considered as targeted, whereas 68.1% (171/251) of the treatments were empirical. The main infection sites were respiratory tract infections (both low tract and pneumonia), bloodstream infections, and urinary tract infections, which were reported as the reason for therapy in respectively 29.9%, 23.5% and 2.4% of the antibiotic treatments for infections.

**Figure 1:**
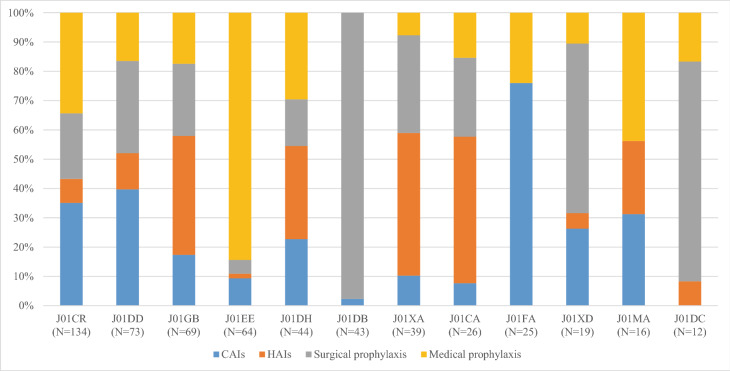
Proportions of treatment by antibiotic drugs and indication of use. Only antibiotic drugs with a frequency of treatment > 1.0% are showed. J01CR: Combinations of penicillins, incl. beta-lactamase inhibitors; J01DD: third generation cephalosporins; J01GB: Other aminoglycosides; J01EE: Combinations of sulfonamides and trimethoprim; J01DH: Carbapenems; incl. derivatives; J01DB: First-generation cephalosporins; J01XA: Glycopeptide antibacterials; J01CA: Penicillins with extended spectrum; J01FA: Macrolides; J01XD: Imidazole derivatives; J01MA: Fluoroquinolones; J01DC: Second-generation cephalosporins.

The top three antibiotic classes were combinations of penicillins including β-lactamase inhibitors (J01CR; 134/587, 22.8%), third-generation cephalosporins (J01DD; 73/587, 12.4%) and aminoglycosides (J01GB; 69/587, 11.8%, Fig. 1).

The pattern of antibiotic use varied by indication: about 98.0% (42/43) of first-generation cephalosporin prescriptions (J01DB) were issued for surgical prophylaxis, followed by second-generation cephalosporins (J01DC; 9/12, 75.0%) and imidazole derivatives (J01XD; 11/19, 57.9%) (Fig. 1).

Combinations of sulphonamides and trimethoprim (J01EE), fluoroquinolones (J01MA) and combinations of penicillins including β-lactamase inhibitors (J01CR) were mainly prescribed for medical prophylaxis (84.4%, 43.8% and 34.3% of the treatments, respectively) (Fig. 1).

CAIs represented the main indication of use for macrolides (J01FA; 19/25, 76.0%), third generation cephalosporins (J01DD; 29/73, 39.7%) and combinations of penicillins including β-lactamase inhibitors (J01CR; 47/134, 35.1%) whereas HAIs were mainly reported for penicillins with extended spectrum (J01CA; 13/26, 50.0%), glycopeptides (J01XA; 19/39, 48.7%) and aminoglycosides (J01GB; 28/69, 40.6%).

### Estimation of DOTs and LOTs

**Figure 2:**
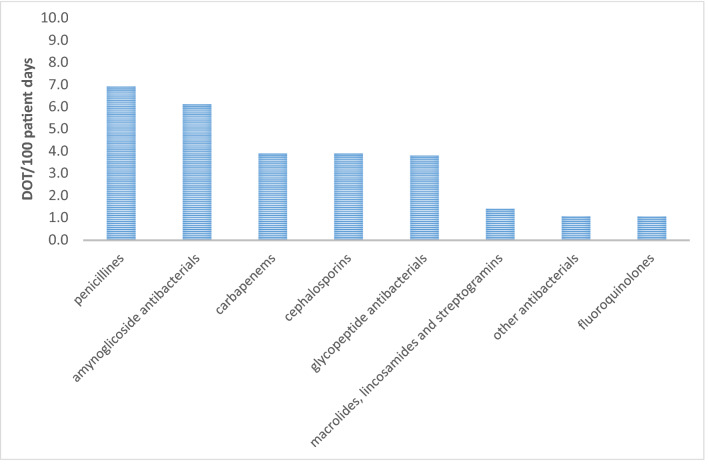
DOT/100 patient days by antibiotic drugs provided for therapeutic purposes. Only antibiotic drugs with more than 1 DOT/100 patient days are shown in the graphic.

DOTs and LOTs were estimated considering 414 inpatient antibiotic treatments, over a total of 9458 inpatient days, retrospectively evaluated considering the 30 days prior to the collection of the point prevalence data on antibiotic use. Overall, we estimated 30.5 DOTs per 100 patient days and a 19.1 LOT per 100 patient days resulting in a DOT/LOT ratio of 1.6 (Table 2). DOT and LOT/100 inpatient days varied significantly by hospital, age, sex, type of ward, and comorbidities. In detail, antibiotic use was higher in males with respect to females, in children aged < 10 years with respect to those aged ≥ 10 years and in the ICUs with respect to medical and surgical wards (Table 2). DOT/100 patient days by type of drug confirmed that penicillins, aminoglycosides, carbapenems and cephalosporins were the antibiotics most frequently prescribed for treatment, with estimated DOT/100 patient days of 7, 6 and 4 (Fig. 2). Targeted therapies had lower DOT/100 patient days than empirical therapies (8.0 DOT/100 vs. 21.0 DOT/100).

**Table 2:** Patient days, DOT–LOT per 100 patient days and DOT/LOT ratio calculated for antibiotics provided for therapeutic purposes by sex, age classes, hospital ward, lenght of hospital stay, comorbidities and participating center.

	Number of patient days	DOT/100 patient days	*P* value_DOT_	LOT/100 patient days	*P* value_LOT_	DOT/LOT ratio
**Sex**						
Male	4779	33.9	< 0.001	20.7	< 0.001	1.6
Female	4653	27.8		17.8		1.6
**Age classes**						
0–11 months	1496	30.1	< 0.001	19.0	< 0.001	1.6
12–23 months	2063	40.3		24.8		1.6
2–4 years	1996	26.5		18.4		1.4
5–9 years	1282	33.5		18.5		1.8
≥ 10 years	2680	25.1		15.7		1.6
**Ward**						
Medical	5362	29.7	< 0.001	18.6	< 0.001	1.6
Surgical	2450	20.6		13.4		1.5
Intensive Care	1736	47.2		28.5		1.7
**Length of hospital stay (days)**						
≤ 7	4154	30.5	0.7	17.9	< 0.001	1.7
08–30	1816	31.3		22.0		1.4
> 30	3578	30.2		18.8		1.6
**Comorbidities**						
Yes	4343	27.3	< 0.001	20.1	< 0.001	1.3
No	4884	33.6		17.7		1.9
**Participating center**						
1	5799	34.6	< 0.001	21.3	< 0.001	1.6
2	1612	40.6		24.9		1.6
3	251	31.1		20.7		1.5
4	1886	9.3		7.1		1.3
**Total**	**9548**	**30.5**		**19.1**		**1.6**

The overall DOT/LOT ratio of 1.6 indicated that the combination antibacterial treatment was common (Table 2).

### Estimation of PDDs and comparison with DDDs

Table 3 shows the median PDDs by age groups for the eight most frequently prescribed treatments. The PDDs increased with age: the ratio between PDDs estimated in children aged ≥ 10 years and PDDs in children aged 0–11 months did greatly vary by antibiotic, and ranged from a minimum of 2 for sulfamethoxazole and trimethoprim, to a maximum of 25 for meropenem. The PDD approached the DDD only in children aged ≥ 10 years: in this age group the PDDs were equal to the DDDs for gentamicin and ceftriaxone, while the median PDD was lower than the DDD for piperacillin/amoxicillin and beta-lactamase inhibitors, sulfamethoxazole and trimethoprim, amikacin and cefazolin. The PDD was higher than the DDD only for meropenem.

**Table 3:** Median PDDs of the most used antibiotic molecules provided for therapeutic purposes by age classes.

Antibiotic molecules	Median PDDs (g) by age classes						
	No. of therapies	DDD (g)	0–11 Months (IQR)	12–23 Months (IQR)	2–4 Years (IQR)	5–9 Years (IQR)	≥ 10 Years (IQR)
Piperacillin and beta-lactamase inhibitors	78	14.0	0.3 (0.07–0.6)	1.0 (0.8–1.6)	3.0 (2.7–4.5)	7.0 (6.0–9.0)	12.0 (9.0–12.0)
Meropenem	71	2.0	0.1 (0.06–0.1)	0.3 (0.2–0.4)	0.8 (0.6–1.3)	1.8 (1.1–1.9)	3.0 (2.1–4.5)
Amoxicillin and beta-lactamase inhibitors	72	3.0	0.3 (0.3–0.3)	0.7 (0.4–0.8)	0.7 (0.4–1.2)	1.5 (1.0–1.8)	2.0 (1.7–2.0)
Sulfamethoxazole and trimethoprim	70	1.9	0.01 (0.01–0.01)	0.3 (0.04–0.4)	0.1 (0.05–0.3)	0.2 (0.09–0.5)	0.3 (0.2–0.3)
Amikacin	64	1.0	0.02 (0.02–0.03)	0.06 (0.05–0.07)	0.2 (0.2–0.6)	0.4 (0.3–0.4)	0.7 (0.5–1.0)
Ceftriaxone	63	2.0	0.3 (0.3–0.3)	0.4 (0.4–0.7)	1.0 (0.5–1.0)	1.5 (1.0–2.0)	2.0 (1.0–2.0)
Cefazolin	61	3.0	0.1 (0.05–0.2)	0.3 (0.2–0.3)	0.7 (0.5–1.0)	1.3 (0.6–1.8)	2.0 (2.0–3.0)
Gentamicin	55	0.2	0.01 (0.004–0.01)	0.01 (0.01–0.02)	0.05 (0.04–0.06)	0.1 (0.08–0.1)	0.2 (0.1–0.3)

## Discussion

Out of more than 800 children included in our study, 47% were treated with at least one antibiotic. This estimate is higher than reported in European hospitalized children, where the point prevalence of antibiotic use was 39.6%^14^. We showed how hospital and patient characteristics, such as age and comorbidities, are related to the use of antibiotics, and other authors have shown that the prevalence of antibiotic use is higher in third-level hospitals where patients generally have more complex conditions^15^. As patient population characteristics are major determinants of antibiotic consumption, risk adjustment should be adopted to compare antibiotic consumption data among hospitals and over-time. In our results, previous surgery was significantly associated with antibiotic use and surgical prophylaxis was the most frequent indication. As previously documented^14–16^, we showed a suboptimal appropriateness of antibiotic surgical prophylaxis; in fact, there was still a proportion of patients treated with non-recommended broad-spectrum active substances, such as III-IV generation cephalosporins.

Another worrying aspect is the duration of surgical prophylaxis; in fact, although a prolonged exposure does not reduce the risk of surgical site infections, it represents a relevant ecological risk^17^. In our study, approximately 77% of the patients undergoing surgical prophylaxis received surgical prophylaxis for more than 24 h. This is in line with previous prevalence surveys, which estimated a proportion of children subject to prolonged surgical prophylaxis (> 1 day) fluctuating between 67 and 78% in Europe^3,16^. These data show that even in the most documented evidence-based area of practice, which recommends a surgical antimicrobial prophylaxis duration of less than 24 h, clinicians tend to overprescribe, and this is a global phenomenon not exclusively limited to Europe^3^. Combinations of sulfonamides and trimethoprim and combinations of penicillins including beta-lactamase inhibitors have been the most widely used active substances for medical prophylaxis, being widely used to prevent opportunistic infections in neutropenic patients^18^. Medical prophylaxis, as well as infection treatments, include a wide range of diseases and it is therefore difficult to identify unambiguous recommendations in patients with very different clinical conditions.

Although point prevalence surveys are useful to assess the pattern of in-hospital antibiotic prescriptions, antibiotic use should be preferably expressed with multiple metrics^19^. We collected DOT, LOT and PDDs as more specific antibiotic indicators for infection treatments with respect to DDDs, which are not recommended for children because doses used are typically much smaller than in adult populations, so this measure underestimates patient exposure to drugs^11^. DOT and LOT have been used to monitor antibiotic use and to assess the impact of antibiotic stewardship programs on improving appropriateness of prescriptions in children^12,13^. We showed that calculation of DOT and LOT by retrospectively reviewing medical records was feasible.

DOT/100 patient days was comparable with antibiotic prescriptions for infections in a secondary pediatric hospital in the Netherlands, where the average DOT/100 in neonatal and pediatric wards was between 23 and 45^20^. Intensive care units were the setting with the highest antibiotic use, with LOT and DOT rates per 100 days of 28.5 and 47.2, respectively. Moreover, a DOT/LOT ratio of 1.6 confirms the frequent resort to multi-therapies found in the prevalence survey where an equal average number of active substances per patient was detected.

Both LOTs and DOTs included the underlying assumption that antibiotic dosing was appropriate. PDDs represented the real prescribed doses of drugs in children. As reported also in a previous study^3^, our study showed that PDD increased with age and approached to DDD only in children aged ≥ 10 years. Although there was no difference between DDDs and PDDs of antibiotic drugs belonging to the class of aminoglycosides and cephalosporins in this age group, a difference was observed for other antibiotic drugs. PDDs lower than DDDs could be explained considering that children’s weight is lower than the standard weight used to compute DDDs, otherwise PDDs higher than DDDs could be reconducted to the patients’ clinical conditions to be treated. It could be the case of meropenem that is frequently used to treat serious healthcare associated bloodstream infections requiring more aggressive dosages.

This multicentre study was conducted in four stand-alone tertiary care children’s hospitals located in Regions in the North, Centre, and South of Italy. Trained investigators collected data regarding antibiotic prescriptions from clinical records minimising the effect of information bias on our study results. Additionally, in contrast to studies that are based on information collected directly from patients medical clinical records, we had no need to restrict the sample size. The study limitations are inherent to the epidemiological methods of our cross-sectional survey, where the main purpose was to describe prescribing patterns in hospitals. The overall rate provided is the average. We did not control for patient case mix, disease incidence, prevalence of different types of infections, variations in resistance levels, institutional factors, all of which can influence antibiotic use patterns^21^.

In conclusion, our results confirm that DOT, LOT and PDD are better alternatives to DDD in children. The availability of electronic clinical records can greatly enhance data accessibility to calculate DOT, LOT and PDD data and use those data within antibiotic stewardship programs, as alternative to DDDs in pediatric population.

## Methods

### Study design and setting

This study was conducted between November and December 2016 and involved four tertiary care children’s hospitals in Italy: Ospedale Infantile Regina Margherita (Turin, in the Region of Piedmont), Ospedale dei Bambini di Brescia (Brescia, in the Region of Lombardy), Bambino Gesù Children’s Hospital (Rome, in the Region of Lazio) and Ospedale Pediatrico Giovanni XXIII (Bari, in the Region of Apulia). These tertiary care children’s hospitals have a total number of beds ranging from a minimum of 157 to a maximum of 607, with an annual number of inpatients ranging from 5700 to 27,000. Pediatric surgeries and pediatric intensive care units are present in all the participating centres; Onco-Haematological Units and Neonatal Intensive Care Units are present in three centres.

### Data collection

The data collection was carried out during a calendar week and involved all children hospitalized on the study days. Data were collected by trained personnel from patients’ paper medical charts. Information collected for each patient included: age, sex, length of hospital stay, and ward type. To assess point prevalence, we collected information about antibiotics administered during the days of data collection. To estimate DOT and LOT, we retrospectively recorded information about antibiotics administered during hospitalization, up to 30 inpatient days prior to the week of data collection. Information on antibiotics included Anatomical Therapeutic Chemical Classification code, ATC J01)^14^, drug brand name, route of administration, dosage, number of doses per day, and indication for antibiotic administration, defined as community-acquired infections (CAIs), hospital-acquired infections (HAIs), medical or surgical prophylaxis. For antibiotics used for treating infections, collected information included also the site of infection and whether microbiological investigations were performed prior to starting antibiotic therapy. The protocol of this study was approved by the Ethical Committee of the Bambino Gesù Children’s Hospital (N° 1238_OPBG_2016) and all methods were performed following relevant guidelines and regulations. Since data were collected by reviewing medical charts and were analyzed anonymously, informed consent was not deemed necessary. The need for informed consent was waived by the Ethical Committee of the Bambino Gesù Children’s Hospital.

All the collected data were uploaded on REDCap (Research Electronic Data Capture) database which is a secure web application for building and managing online surveys and databases, available at no charge to not-for-profit institutions^22^. All the data were analyzed anonymously.

### Statistical analysis

Patients were described according to demographic and clinical factors. Collected data were presented as count and proportions (categorical data) or median and interquartile range (IQR, continuous data). The wards where the patients were hospitalized were categorized as medical (e.g. general neonatal and pediatric wards, oncology and hematology), surgical (e.g. neonatal surgery, pediatric surgery, cardiac surgery, neurosurgery, ENT, orthopedics) and intensive care units (ICUs) (e.g. pediatric intensive care unit, neonatal intensive care unit, and cardiac intensive care unit).

The metrics we calculated included point prevalence of antibiotic use, DOTs, LOTs and PDDs. The prevalence of antibiotic use was calculated as the ratio between the number of patients under treatment with at least one antibiotic on the survey day, and the total number of hospitalized patients. Prevalence of use was computed by age group (0–11 months, 12–23 months, 2–4 years; 5–9 years; ≥ 10 years), sex, hospital ward, lenght of hospital stay, comorbidities, surgical procedures during hospitalization, participating centre and indication. Comparisons among groups were conducted using the Chi-squared test.

Antibiotic drugs were classified according to the Anatomical Therapeutic Chemical Classification code (ATC: J01); a percentage distribution of antibiotic drugs substances by indication of use was computed considering the antibiotic prescription administered on the survey date and those received in the previous 30 days.

DOTs and LOTs were computed on treatments^20^, considering the 30-days study period. For each patient, DOTs were computed summing up the duration (in days) of each antibacterial drug received for treating infections. LOTs were the number of days that a patient received an antibacterial drug irrespective of the number of different drugs^12^. DOTs/100 patient days and LOTs/100 patient days were computed stratifying for sex, age classes, ward type, type of active substances and targeted *versus* empirical therapy; groups comparisons were performed using Student’s t-test and one way ANOVA test. PDDs were defined as the observed dose received by each patient in grams and was calculated for the most frequently used antibiotics. Due to the extremely large differences in dosing in pediatrics, ranging from pre-term neonates to teenagers, median PDDs, and the respective IQRs, were estimated and stratified into five age groups (1, 0–11 months; 2, 12–23 months; 3, 2–4 years; 4, 5–9 years; 5, ≥ 10 years), as previously described^3^. All statistical analyses were conducted using STATA 13 (Stata Corporation, College Station, Texas, USA).
